# Supplementing a *Bacillus*-based direct-fed microbial improves feed efficiency in lactating dairy cows

**DOI:** 10.1093/tas/txae110

**Published:** 2024-07-23

**Authors:** Marta Terré, Norbert Prat, Daniel Sabrià, Oscar Queiroz, Jens N Joergensen, Giuseppe Copani, Bruno I Cappellozza

**Affiliations:** Department of Food Production, IRTA, Torre Marion, Caldes de Montbui, Spain; Estació de Vacum de Monells, IRTA, Monells, Spain; Department of Food Production, IRTA, Torre Marion, Caldes de Montbui, Spain; Estació de Vacum de Monells, IRTA, Monells, Spain; Department of Food Production, IRTA, Torre Marion, Caldes de Montbui, Spain; Estació de Vacum de Monells, IRTA, Monells, Spain; Animal Biosolutions, Novonesis, Hørsholm, Denmark; Animal Biosolutions, Novonesis, Hørsholm, Denmark; Applied R&D, Novonesis, Hørsholm, Denmark; Animal Biosolutions, Novonesis, Hørsholm, Denmark

**Keywords:** *Bacillus* spp, direct-fed microbial, feed efficiency, milk yield

## Abstract

This experiment was conducted to evaluate the effects of feeding a *Bacillus*-based direct-fed microbial (**DFM**) on performance and nutrient digestibility of lactating dairy cows. Seventy-six lactating (42 ± 6 days in milk [**DIM**]) Holstein–Friesian primiparous and multiparous cows were enrolled to a 16-wk experiment. Cows were blocked by lactation number and DIM and within blocks, assigned to 1 of the 2 treatments: 1) basal partial-mixed ration (**PMR**) without DFM addition (*n* = 38; **CON**) or 2) basal PMR with the addition of 3 g/head/d of a DFM containing *B. licheniformis* 809 and *B. subtilis* 810 (*n* = 38; BOVACILLUS, Chr. Hansen A/S, Hørsholm, Denmark; **DFM**). The DFM was mixed in a protein-based pellet, whereas the CON group was fed the same pellet without DFM (0.6 kg/cow/d). The PMR contained (dry matter [**DM**] basis) 50% of forage and 48% of a concentrate feed based on corn meal, soybean meal, wheat meal, wheat middlings, and a mineral–vitamin premix, with the remaining part of the diet being represented by the pellet used as a carrier for the treatments (CON and DFM). Dry matter intake (**DMI**), milk yield, and production efficiency were recorded daily, whereas milk protein and fat concentrations were recorded using electronic milk meters. An additional milk sample was collected every second week of the study for milk composition. On week 15 of the study, fecal samples were collected from each cow for apparent nutrient digestibility calculation. All data were analyzed using the MIXED procedure of SAS (version 9.4; SAS Inst. Inc., Cary, NC). No treatment effects were observed on cow final body weight, daily **DMI**, milk yield, energy-corrected milk (**ECM**), ECM efficiency, milk composition (yield or content), and somatic cell count (**SCC**) (*P* ≥ 0.12). However, cows fed DFM had a greater feed and N efficiency (*P* ≤ 0.03) compared to cows fed CON. Moreover, DM digestibility tended to be greater for DFM-fed cows when compared to CON (*P* = 0.10), whereas no further nutrient digestibility differences were observed (*P* ≥ 0.24). In summary, supplementing a DFM containing *Bacillus licheniformis* and *B. subtilis* benefited feed efficiency of lactating dairy cows fed a PMR, while also tending to improve the digestibility of DM.

## Teaser

**•** Feed efficiency is a key trait impacting the profitability of dairy operations

**•** Adding the *Bacillus* spp. improved feed efficiency, N and fat efficiency in lactating Holstein–Friesian dairy cows

## Introduction

Direct-fed microbials (**DFM**) have been fed to the dairy calves and cows to support overall health and performance of the herd (Nocek and Kautz, 2006; Nalla et al., 2022). Regarding performance, not only growth rates and milk production in calves and cows, respectively, can be optimized by DFM feeding, but also feed efficiency improvements have been reported (Valldecabres et al., 2022; Oyebade et al., 2023). The underlying mechanisms involved in this response are likely to be multifactorial, as different modes of action to support systemic and gastrointestinal tract health have been demonstrated, microbiota modulation (Lamontagne et al., 2023), as well as a better feed and nutrient utilization in the rumen and lower GIT could result in greater feed and milk production efficiency that, in turn, would benefit the profitability of the dairy operations (Connor, 2015).

Based on the aforementioned rationale and goal, it would be ideal to feed a bacterial DFM that can i) support the GIT health, ii) stimulate the production and release of digestive enzymes that ultimately improve nutrient utilization by the host, and iii) improve performance traits in dairy cattle, including milk production efficiency. Hence, one feasible bacterial DFM candidate that can accomplish these health and production goals is Bacilli, a spore-forming bacteria that can survive and thrive through most, if not all, challenges faced during feed preparation (Cappellozza et al., 2023a) and within the GIT of the host (Bernardeau et al., 2017). Moreover, *Bacillus* spp. have been shown to support the GIT health through different mechanisms, including in vitro mucin production (Santano et al., 2020) and biofilm formation (Segura et al., 2020), but also to increase in vitro dry matter (**DM**), neutral detergent fiber (**NDF**), and starch digestibility (Pan et al., 2022; Cappellozza et al., 2023b). However, few in vivo studies have evaluated the combination of *Bacillus* strains on performance and nutrient digestibility of early-lactating dairy cows. Hence, we hypothesized that feeding a DFM containing *Bacillus* spp. would improve feed efficiency and nutrient digestibility of Holstein–Friesian lactating dairy cows. Therefore, our objective was to evaluate the effects of supplementing a *Bacillus*-based DFM on performance and nutrient digestibility of lactating Holstein–Friesian cows.

## Experimental Procedure

This experiment was conducted from September 2021 to December 2022 at the Estació de Vacum de Monells (EVAM), Institute of Agrifood Research and Technology (IRTA), Girona, Spain. All procedures were approved by the Animal Care Committee of the Government of Catalonia (# 11450).

On day 0 of the study, 76 lactating (42 ± 6 DIM) Holstein–Friesian primiparous (*n* = 34) and multiparous (*n* = 42) cows were enrolled to a 16-wk experimental period, comprising of 1 wk of adaptation and for covariates and 15 wk of treatment administration and individual data collection. Throughout the experimental period, all cows were fed a partial-mixed ration (**PMR**) and on top of that offered 0.6 kg/d/cow of a protein-based pelleted concentrate in the milking parlor. The PMR contained 50.0% of forages, including ryegrass hay, wheat silage, corn silage, ryegrass silage, and alfalfa hay (DM basis), and a concentrate feed added at 48.0% of the diet. The concentrate feed contained corn meal, soybean meal, wheat meal, wheat middlings, and a mineral–vitamin premix. The diet was formulated using the ration formulation Bestmix software (Maldegem, Belgium) to meet or exceed the nutritional requirements of lactating dairy cows producing at least 35 kg of milk/d. Table 1 reports the composition and nutritional profile of the diet utilized throughout the experiment. According to the calving schedule, cows were blocked by parity and DIM, and within blocks established by a week of study enrollment, assigned to 1) basal PMR and protein concentrate without the addition of a DFM (*n* = 38; **CON**) or 2) basal PMR and protein concentrate with the addition of 3 g/head/d of a DFM containing a mixture of *Bacillus licheniformis* 809 and *B. subtilis* 810 (BOVACILLUS; 3.2 × 10^9^ colony forming units/g; Chr. Hansen A/S, Hørsholm, Denmark; *n* = 38; **DFM**). The DFM was mixed in a protein-based pelleted concentrate and offered at 0.6 kg/cow/d to the DFM group, whereas CON cows were also fed 0.6 kg of the same protein pellet daily without the DFM.

**Table 1.** Composition and nutritional profile (±SEM) of the PMR offered to the cows during the experiment^1^

Ingredient	Inclusion, % DM
Concentrate	48.0
Corn meal	21.7
Soybean meal	13.7
Wheat meal	10.5
Wheat middlings	0.47
Calcium carbonate	0.85
Sodium chloride	0.29
Magnesium oxide	0.24
Mineral–vitamin premix^2^	0.21
Forage	50.0
Ryegrass hay	14.7
Wheat silage	14.1
Corn silage	11.1
Ryegrass silage	8.3
Alfalfa hay	1.8
Soybean pellet	2.0
Nutritional profile	% or % DM
Dry matter	58.1 ± 2.13
Crude protein	15.5 ± 0.89
Neutral detergent fiber	33.4 ± 2.43
Acid detergent fiber	19.3 ± 2.42
Starch	28.5 ± 2.95
Ether extract	2.3 ± 0.44
Ash	7.7 ± 1.94

^1^The study lasted 16 wk, comprising 1 wk of adaptation and data collection for covariates followed by 15 wk of treatment (CON and DFM) administration and data collection.

^2^14.6% Ca, 0.03% Na, 4.48% Mg, 2,250,000 UI/kg Vitamin A, 8,800 mg/kg Vitamin E, 665,000 UI/kg Vitamin D3, 40 mg/kg Co, 30,000 mg/kg Zn, 150 mg/kg Se, 20,000 mg/kg Fe, 250 mg/kg I, 30,000 mg/kg Mn, 5,000 mg/kg Cu, 1,500 mg/kg Butylhydroxytoluene, 279,949 mg/kg sepiolite.

All cows were fed the PMR in amounts to ensure ad libitum intake with at least 5% of daily feed refusal using feed bunkers with automated individual feeding recording (MooFeeder, MooSystem, Cortes, Spain). The PMR was prepared using a chopping–mixing wagon and delivered in the feed bunks twice per day (approximately at 0800 and 1900 hours) using a tractor with a fodder unloading bucket. Each morning, the feed bunkers were emptied (approximately at 0700 hours) before the first feeding of the day. The concentrate feed was offered separately in the milking parlor (MooSystem) during the milking twice daily. Moreover, 300 g of the pelleted soybean supplement, containing or not DFM, were offered in the milking feeders twice daily. The counts of the spores were performed postpelletization following the procedures and methodologies described by Cappellozza et al. (2023a). In all the samples, the recovery was adequate considering the initial inclusion (7.2 log_10_ CFU/g pellet).

All nutritional analyses of the feedstuffs were performed at the ELAB laboratory (Vic, Barcelona, Spain). The DM content of the PMR was analyzed twice per week. An additional sample of the PMR was pooled per week and analyzed monthly for determination of its nutritional profile. Ash content was determined by ignition at 550 °C for 4 h, crude protein (**CP**) was analyzed by an automated Kjeldahl procedure (Foss, Hillerød, Denmark), and ether extract was determined following the methodology described by AOAC (1990; method #920.39). Ash-free NDF was analyzed according to Chai and Udén (1998), and acid detergent fiber (**ADF**) determined according to Van Soest et al. (1991). Lastly, starch content was determined using the polarimetric method reported by the Commission Directive 1999/79/EC.

Cows were milked twice daily at 0700 and 1830 hours and individual milk yield was recorded daily during the entire experiment (Tecnozoo, Cortes, Spain). Daily milk yield and fat and protein contents were recorded using electronic milk meters (AfiMilk, Afikim Ltd, Kibbutz Afikim, Israel) and an online analysis of milk components concentrations in the milking parlor (AfiLab system, Afikim Ltd). Each of these components uses near-infrared spectroscopy (**NIR**) for online milk analysis (Tsenkova et al., 1999) that has been validated in previous publications (Kaniyamattam and De Vries, 2014). These data were considered for weekly productive performance of the cows in both CON and DFM groups. Morning and afternoon milk samples were collected every 2 wk during the entire experimental period. Milk samples were analyzed in a commercial laboratory (Laboratori Interprofessional Lleter de Catalunya—Associació Interprofessional Lletera de Catalunya, Cabrils, Spain) for lactose, solid nonfat (**SNF**), and milk urea-N (**MUN**) with an infrared technology (MilkoScan 7; Foss Iberia S.A., Barcelona, Spain), and SCC were evaluated through the near-infrared spectroscopy (Fossomatic; Foss Iberia S.A.) technique. Milk composition was averaged by sampling week and milk lactose and SNF were determined by multiplying the weekly average milk yield with the concentration of lactose and SNF from the test day of each cow. Data for individual cow total dry matter intake (**DMI**) was collected daily, pooled, and evaluated per week, whereas feed efficiency was determined by dividing milk yield by total DMI. Moreover, energy-corrected milk (**ECM**) was calculated using published equations (Tyrell and Reid, 1965), whereas ECM efficiency was determined by dividing ECM by DMI. Lastly, N efficiency calculated as (milk nutrient output/by nutrient intake) × 100 was reported for both treatments, and cow body weight (**BW**) was recorded in the beginning and end of the study.

On week 15 of the study and for 3 d, 300 g of fecal spot samples were manually collected directly from the rectum of the cow at 0500, 1100, and 1700 hours (day 1), 0700, 1300, and 1900 hours (day 2), and at 0900, 1500, and 2100 hours (day 3) from 23 and 22 cows from CON and DFM, respectively, for apparent nutrient digestibility analysis. Fecal samples were stored at −20 °C and after thawing, samples were dried at 60 °C, pooled within cow, milled, and analyzed for DM, ash, CP, NDF, and acid insoluble ash (**AIA**) as previously described. Fecal excretion was calculated from the total intake of AIA and fecal AIA content, assuming 100% of AIA recovery (Van Keulen and Young, 1977). The apparent digestibility of DM, organic matter, CP, NDF, ADF, and starch was calculated from recorded individual feed intake and estimated excretion of each nutrient.

The sample size was determined with the UBC Power Calculator (https://www.stat.ubc.ca/~rollin/stats/ssize/n2.html) using an alpha of 0.05 and power of 0.80 to detect 2.5% difference in milk yield and 2.7% difference on feed efficiency. All data were analyzed using the PROC MIXED of SAS (version 9.4; SAS Inst. Inc., Cary, NC, USA), the Satterthwaite approximation to determine the denominator df for the test of fixed effects, and block as random variable. Data obtained on week 1 were used as covariates and production data were analyzed using the repeated statement of SAS, considering week as the repeated term, and cow(treatment) as the subject. The first-order autoregressive structure was chosen as it provided the lowest Akaike Information Criterion. Fixed effects included treatment, week, and the resulting treatment × week interaction. Somatic cell counts were initially log-transformed to meet the requirement of the normal distribution assumptions and transformed-back (as × 1,000 cells/mL) for reporting in the manuscript. Lastly, nutrient digestibility data was analyzed and considered the main effect of treatment only. All data were reported as least square means and covariately adjusted to the values obtained on week 1. Significance was set at *P* ≤ 0.05, tendencies denoted if 0.05 < *P* ≤ 0.10, and results were reported according to the main effects if no interactions were significant.

## Results

During the covariate period, no differences were observed for any of the productive and milk composition variables (*P* ≥ 0.15; data not shown), the only exception being milk fat content (*P* = 0.04; 3.18% vs. 3.39% for CON and DFM, respectively; SEM = 0.127). Nonetheless, all productive data collected on week 1 were significant covariates (*P* < 0.001; data not shown).

For all the productive traits, no treatment × week interactions were observed (*P* ≥ 0.19), so only main effects will be reported herein. No significant differences were observed on DMI, milk yield, ECM, milk composition (content or yield) of lactating dairy cows receiving CON or DFM (*P* ≥ 0.12; Table 2). Nonetheless, feed efficiency (kg milk/kg DM consumed) was greater for DFM vs. CON cows (*P* = 0.03; Table 2), whereas no further differences were observed on ECM production efficiency (*P* ≥ 0.16). Lastly, N intake was lower for DFM-supplemented cows (*P* = 0.01), whereas N and efficiency were greater when compared with CON cows (*P* ≤ 0.01; Table 2).

**Table 2.** Performance results of lactating dairy cows supplemented or not (CON; *n* = 38) with a *Bacillus*-based DFM (*n* = 38)^1^

Item	Treatments		SEM	*P* value
	CON	DFM		
Cow production				
Initial body weight, kg	708.5	708.1	15.53	0.99
Final body weight, kg	709.2	707.9	13.03	0.94
DMI, kg/d	25.0	24.5	0.31	0.16
Milk yield, kg/d	38.0	38.3	0.50	0.57
Feed efficiency, kg milk/kg DMI	1.54	1.59	0.020	0.03
ECM yield, kg/d^2^	38.0	38.2	0.44	0.79
ECM efficiency, kg ECM/kg DMI	1.56	1.59	0.023	0.18
Yield, kg/d				
Protein	1.24	1.27	0.018	0.35
Fat	1.32	1.31	0.024	0.55
Lactose	1.88	1.89	0.040	0.91
Solid nonfat	3.36	3.39	0.022	0.28
Content, %				
Protein	3.30	3.32	0.032	0.45
Fat	3.54	3.46	0.055	0.12
Lactose	4.95	4.93	0.023	0.35
Solid nonfat	8.85	8.86	0.033	0.83
MUN, mg/L	211.0	218.0	7.41	0.31
SCC, ×1,000 cells/mL	337.7	444.8	36.72	0.49
N parameters				
Intake, kg/d	3.87	3.80	0.054	0.01
Efficiency, %^3^	32.6	33.8	0.60	<0.001

^1^CON = basal PMR offered to ensure ad libitum intake throughout the trial; DFM = CON diet with the addition of 3 g/head/d of a *Bacillus*-based DFM (BOVACILLUS).

^2^ECM (kg/d) = 0.327 × milk yield (kg) + 12.95 × fat yield + 7.65 × protein yield (Tyrell and Reid, 1965).

^3^Calculated as milk nutrient yield divided by nutrient intake in the basal diet.

For apparent nutrient digestibility, cows fed DFM tended to have a greater DM digestibility (*P* = 0.10) on week 16 of the study, but no further differences were observed in the digestibility of the other nutrients (*P* ≥ 0.24; Table 3).

**Table 3.** Nutrient digestibility of lactating dairy cows supplemented or not (CON) with a *Bacillus*-based DFM^1^^,^^2^

Item	Treatments		SEM	*P* value
	CON	DFM		
Nutrient digestibility, %				
Dry matter	71.4	72.9	1.14	0.10
Organic matter	74.4	74.9	1.19	0.59
Neutral detergent fiber	56.5	55.8	1.98	0.64
Acid detergent fiber	55.7	56.5	2.17	0.63
Starch	96.5	97.1	0.38	0.24
Ether extract	58.6	59.1	3.02	0.91

^1^CON = basal PMR offered to ensure ad libitum intake throughout the trial; DFM = CON diet with the addition of 3 g/head/d of a *Bacillus*-based DFM (BOVACILLUS).

^2^For nutrient digestibility, 23 and 22 cows from CON and DFM groups were sampled, respectively.

## Discussion

The main goal of the present experiment was to evaluate the effects of feeding a *Bacillus*-based DFM containing *B. licheniformis* 809 and *B. subtilis* 810 on performance and apparent total-tract nutrient digestibility of lactating Holstein–Friesian dairy cows fed a PMR containing 50% forages. Previous studies in the literature have reported the stability feature of *Bacillus* spp. when added to different feed matrices (Cappellozza et al., 2023a), but also the beneficial effects of feeding *Bacillus* spp. to the dairy cattle herd, including improvements in milk yield (Sun et al., 2013; Souza et al., 2017; Cappellozza et al., 2024), fat- and energy-corrected milk yield (Sun et al., 2013; Oyebade et al., 2023a), yield and content of milk components (Cappellozza et al., 2024; Oyebade et al., 2023a), health parameters (Segura et al., 2020; Lucey et al., 2021), calf growth performance (Kowalski et al., 2009; Magalhaes et al., 2024), reduction in somatic cell counts (Sun et al., 2013), beneficial alterations in the rumen microbiota profile (Sun et al., 2016; Lamontagne et al., 2023), metabolomics (Oyebade et al., 2024), rumen fermentation traits (Sun et al., 2016; Lamontagne et al., 2023; Cappellozza et al., 2024), rumen pH, as well as greater *in sacco* DM and NDF digestibility (Sun et al., 2013; Ushakova et al., 2013). Moreover, previous in vitro work demonstrated that adding the same combination of *Bacillus* spp. evaluated herein into commercial dairy TMR sampled from nine different locations in the United States resulted in a greater in vitro gas production, DM, and NDF degradability over a 48-h period (Cappellozza et al., 2023b).

In dairy operations, feed costs may account up to 60% of total production costs (Bach, 2012) and alternatives that optimize the utilization of feedstuffs included in the diets of dairy cows will likely benefit overall profitability of dairy operations. One of the strategies to increase the utilization of dietary feedstuffs is to promote some production traits in the herd, such as feed efficiency in lactating cows from the same herd under the same management (Connor, 2015). Our results demonstrate that feeding a combination of *B. licheniformis* 809 and *B. subtilis* 810 improved feed efficiency by 3.2% (+50 g of milk/kg of DM consumed), even though no significant treatment differences were observed in milk yield and total DMI. Arndt et al. (2015) suggested that improvements in feed efficiency were not likely to be observed in situations where milk production was considered to be already adequate, partly due to the losses on digestible energy associated with high DMI and milk production (NRC, 2001). Nonetheless, our feed efficiency values are comparable to previous authors evaluating dairy cows either throughout a full 305-d lactation (1.61 kg/kg; Vallimont et al., 2011) or mid- to late-lactating cows (1.43 kg/kg; Arndt et al., 2015). Our results indicate that even when high-producing, well-managed lactating dairy cows are evaluated, *Bacillus*-based DFM supplementation could be a feasible alternative to promote the efficiency of the dairy cow herd, without causing changes in nutrient reserves and, therefore, body condition score, energy balance, and BW of the herd (Spurlock et al., 2012; Vallimont et al., 2013). Moreover, supplementation of the *B. licheniformis* 809 and *B. subtilis* 810 to Swedish-Red and Holstein–Friesian lactating dairy cows under the similar days in milk as herein has improved milk yield (+ 1.2 kg/d) and feed efficiency (+ 40 g milk/kg) when fed for an 84-d experimental period (Cappellozza et al., 2024).

Another interesting and novel finding of the present experiment was the fact that DFM supplementation led to a greater N and fat efficiency when calculated as milk nutrient output divided by nutrient intake through the basal diet. Improvements in N use efficiency can reduce N excretion from dairy production settings, likely alleviating the amount of N lost to the environment (Eggleston et al., 2006; Sajeev et al., 2018; de Souza et al., 2023). One of the main features of *Bacillus* strains is their ability to produce a wide range and quantity of enzymes of interest for livestock production, including cellulase, xylanase, amylase, protease, and lipase (Schallmey et al., 2004; Luise et al., 2022). Therefore, it is hypothesized that when *Bacillus* spp. are fed to livestock animals, an improvement in nutrient digestibility might be observed, leading to a greater herd performance, feed, and nutrient efficiency. In fact, we reported a tendency of greater DM digestibility in lactating dairy cows fed the *Bacillus*-based DFM, corroborating with previous in vitro (Pan et al., 2022; Cappellozza et al., 2023b) and in vivo work (Sun et al., 2013). Nonetheless, the lack of treatment effects on the digestibility of other nutrients has also been reported herein and when *Bacillus* spp. were fed diets containing more than 50% forages (Souza et al., 2017; Cappellozza et al., 2024). Based on these differences between experiments, it can be speculated that the composition and nutrient profile of the diets might impact the magnitude of responses regarding herd performance and nutrient digestibility following a *Bacillus*-based DFM supplementation (Krehbiel et al., 2003; Seo et al., 2010; McAllister et al., 2011), as bacilli enzymatic expression might be stimulated by different nutrients, in a mechanism called “induction effect”, as reported by others (Beauregard et al., 2013; Tian et al., 2021). Lastly, it cannot be excluded the potential effects of *Bacillus* spp. supplementation on promoting the growth and activity of carbohydrate-degrading bacteria (Sun et al., 2013; Lamontagne et al., 2023; Linde et al., 2023) that could optimize the utilization of nutrient by the rumen microorganisms, leading to a reduction in rumen ammonia concentration (Dias et al., 2022) and overall greater nutrient utilization efficiency in the gastrointestinal tract of the animals.

In summary, feeding a *Bacillus*-based DFM containing *B. licheniformis* and *B. subtilis* improved feed efficiency by 50 g of milk/kg of DM consumed, as well as N and fat use efficiency, while also tending to improve apparent total-tract DM digestibility. Additional studies are warranted to understand how different dietary composition and nutritional profiles impact the productive responses and nutrient utilization in dairy cows fed a *Bacillus*-based DFM.
